# Ketamine in Trauma: A Literature Review and Administration Guidelines

**DOI:** 10.7759/cureus.48099

**Published:** 2023-11-01

**Authors:** Kristen Reede, Reid Bartholomew, Dana Nielsen, Mentor Ahmeti, Khaled Zreik

**Affiliations:** 1 General Surgery, University of North Dakota School of Medicine and Health Sciences, Grand Forks, USA; 2 Trauma Surgery, University of Tennessee Health Science Center, Memphis, USA; 3 Surgery, Sanford Medical Center, Fargo, USA; 4 Surgical Critical Care, Sanford Medical Center, Fargo, USA

**Keywords:** ketamine utility in trauma, ketamine administration, ketamine dosing, pain control, trauma, dissociation, ketamine, procedural sedation and analgesia

## Abstract

Ketamine is a phencyclidine (PCP) derivative, which primarily acts as a noncompetitive N-methyl-D-aspartate (NMDA) receptor antagonist. Ketamine serves as an analgesic and a dissociative sedative that produces potent analgesia, sedation, and amnesia while preserving spontaneous respiratory drive. It is rapidly gaining acceptance in the management of pain as multiple studies have demonstrated its reliable efficacy and a wide margin of safety. This article reviews some of these studies, the history of ketamine, and its pharmacological and pharmacokinetic properties. The article also discusses the use of ketamine in the trauma setting, including joint reductions, procedures, sedation, and pain control, as well as dosing recommendations.

## Introduction and background

Physical pain caused by trauma can be a challenging problem for professionals involved in trauma care. Pain may not be controlled for several reasons, such as being underappreciated by physicians, hemodynamic instability concerns, respiratory depression, and addiction fears. Cohen et al. discussed the importance of adequate pain control in the trauma setting and how persistent and severe pain can alter the nervous systems’ anatomical and physiological functions [1]. They state that repeated incoming stimuli to the nervous system can cause neuroplasticity, which can lead to the development of chronic pain. The stress response after trauma results in the release of cytokines and acute phase reactants, elevated levels of catecholamines, adrenocorticotropic hormones, activation of the renin-angiotensin system, impaired coagulation process, and an altered immune response. In patients with inadequately treated acute pain, this stress response is exacerbated and can further potentiate adverse effects trauma has on normal physiology including ventilation, hemodynamic stability, and gastrointestinal and renal function. Further compromise of these already impaired processes can result in increased morbidity and mortality [1].

Opioids are considered the cornerstone for the treatment of severe pain in emergency trauma patients [2]. These medications have some disadvantages. Opioids have both short- and long-term risks including nausea, constipation, respiratory depression, tolerance, dependence, abuse, and overdose. Thousands of Americans die each year because of the opioid epidemic. Preliminary data shows that in 2021, over 100,000 deaths were the result of drug overdoses in the United States. Opioids were involved in over 78% of those overdoses, and prescription opioids accounted for 19% [3].

Medical professionals often prescribe opioids to treat pain. It has been demonstrated that over 50% of patients will receive a narcotic prescription upon discharge after traumatic injuries [4,5]. As the use of narcotics increases, trauma centers are taking care of an increasing number of patients who are on chronic narcotic therapy. Studies have demonstrated that 20% of injured patients reported having been prescribed narcotics and/or benzodiazepines before admission. These patients had statistically significantly longer ICU length of stay, longer total hospital length of stay, and increased requirements for mechanical ventilation [6].

Analgesic options that reduce opioid use while managing acute pain are being sought by trauma surgeons to combat this epidemic as it is essential for optimal outcomes. The purpose of this article is to review the use of ketamine in trauma patients.

## Review

History

**Figure 1:**
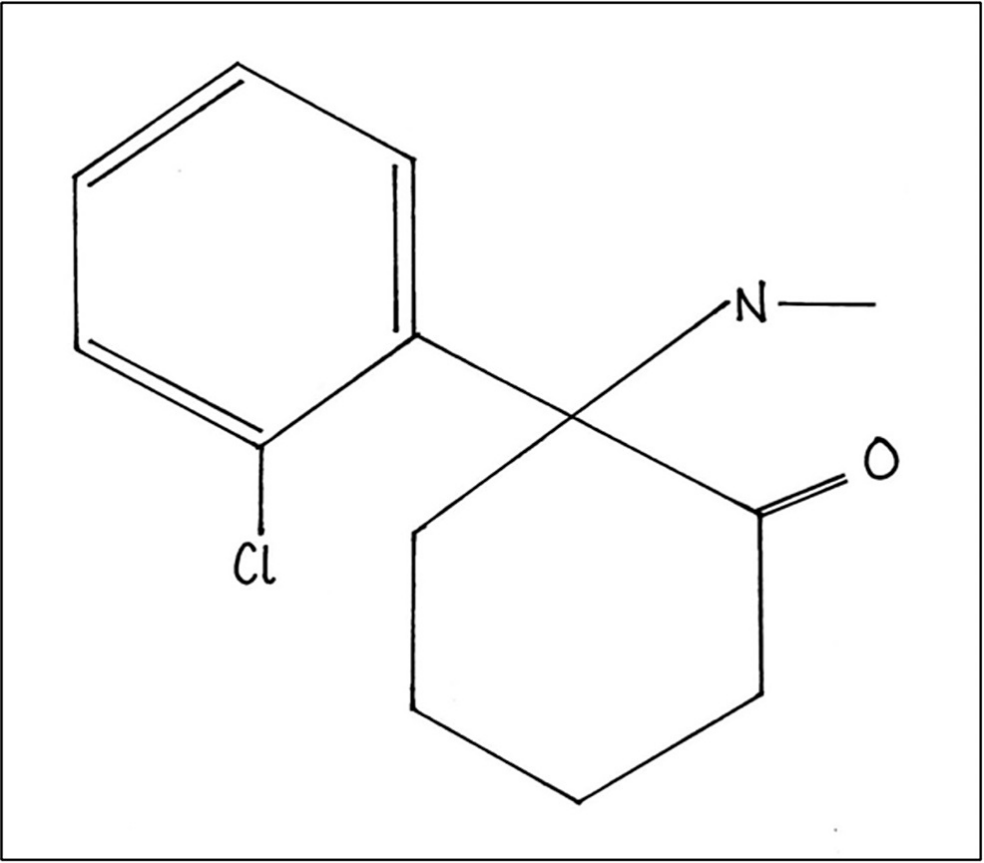
Chemical structure of ketamine Image credits: This drawing was created by Dana Nielsen, a co-author.

The history of ketamine began in the 1950s when phencyclidine (PCP) was first synthesized as a general anesthetic by the pharmaceutical company Parke-Davis in Detroit, Michigan. It was described as a potent general analgesic that did not cause cardiovascular or respiratory depression; however, it was known to have a high rate of side effects, including frequent hallucinations, postoperative delirium, and confusion [7]. Attempts were made to identify an alternative to PCP that retained the anesthetic result without serious psychotomimetic side effects. In 1962, Calvin Stevens was able to accomplish this by synthesizing ketamine, a PCP derivative (Figure 1) [8]. The first human administration of ketamine was conducted by Corseen and Domino in 1964 to volunteer prisoners at the Jackson Prison in the state of Michigan. In 1970, the Food and Drug Administration officially approved it due to its sympathomimetic properties and its wide margin of safety. In the Vietnam War, it was often used as a field anesthetic by soldiers. Due to concerns over the so-called psychedelic effects and the arrival of new intravenous hypnotics such as propofol, there was a decrease in the use of ketamine. There was also an increased prevalence of abuse of the drug, which led to ketamine being placed among the class III substances of the US Controlled Substance Act in 1999 [7]. Nevertheless, the interest in ketamine continued, and additional research allowed a further understanding of its mechanism of action and discovered further utilities for the drug. The clinical application of ketamine continues to expand and includes intubation, procedural sedation, chemical anxiolysis in acute agitation, neuropathic pain, migraine headaches, adjunctive treatment of major depressive disorders, and general anesthesia [9-12].

Pharmacology

**Figure 2:**
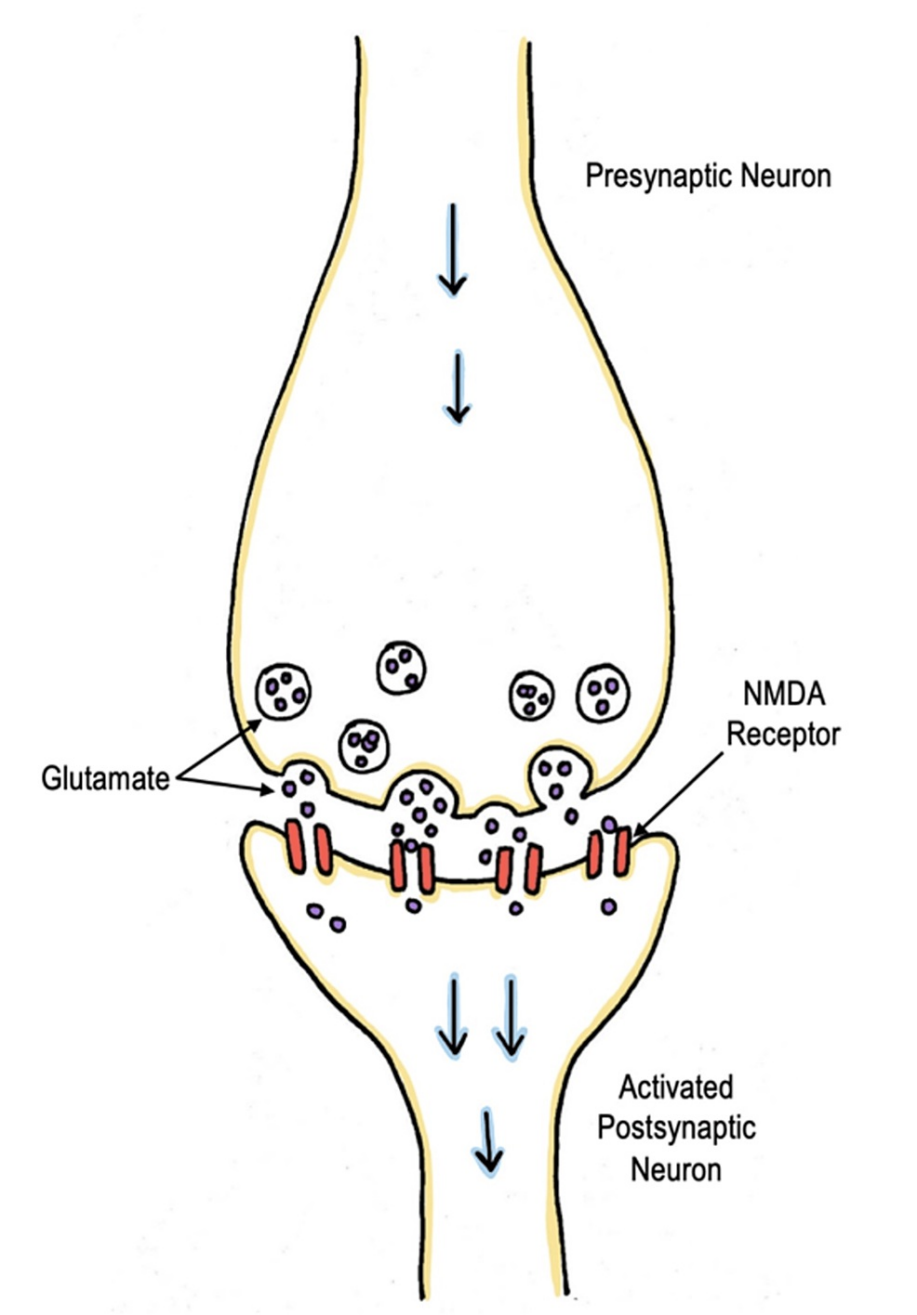
Normal physiology Glutamate binds to the NMDA receptor, causing the membrane to depolarize and ultimately activate the neuron. Image credits: This drawing was created by Dana Nielsen and Kristen Reede, co-authors. NMDA: Noncompetitive N-methyl-D-aspartate.

**Figure 3:**
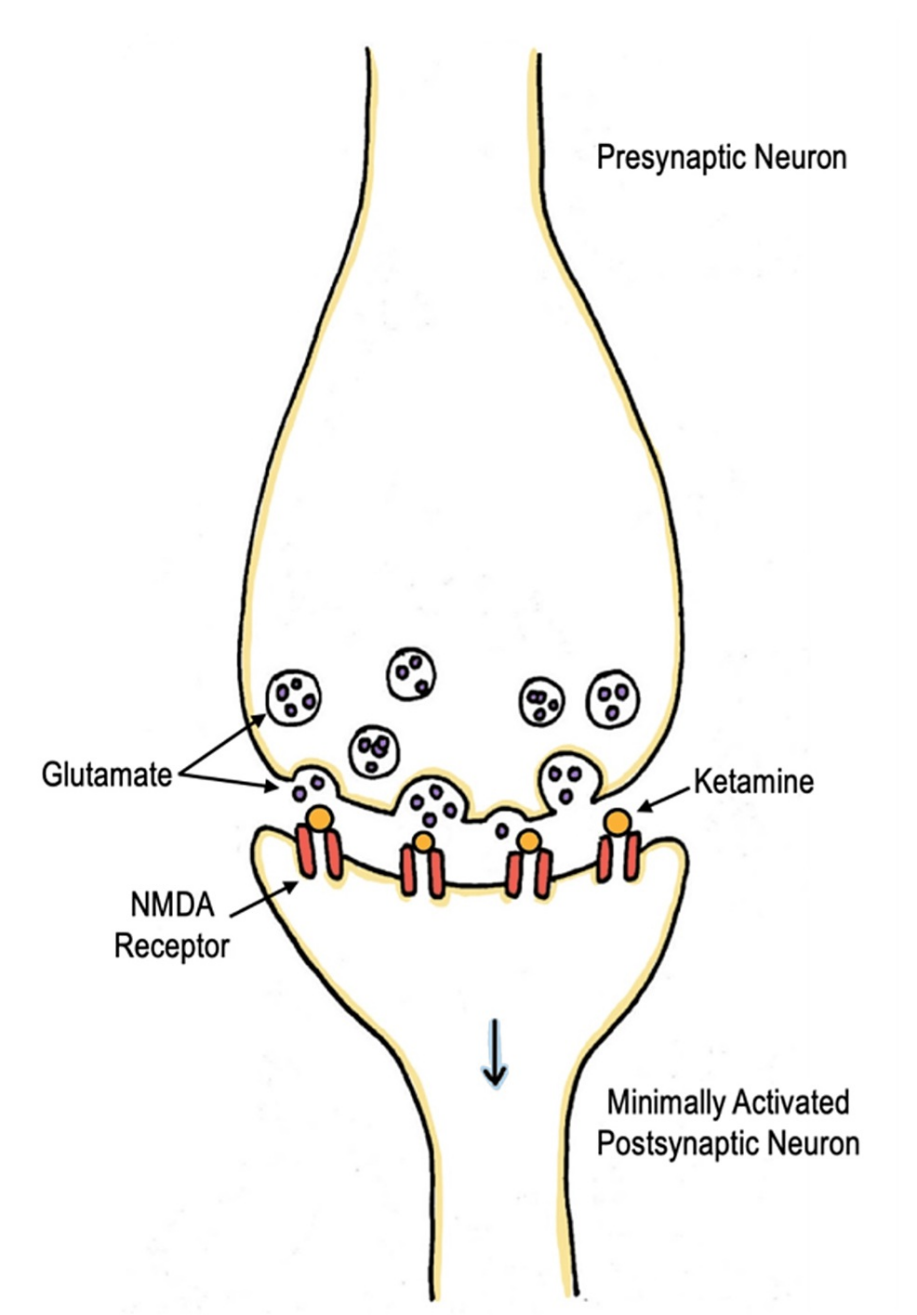
Mechanism of ketamine at anesthetic and dissociative dosing Ketamine blocks the NMDA receptor, preventing the receptor from being activated by glutamate and decreasing the excitability of the neuron. Image credits: This drawing was created by Dana Nielsen and Kristen Reede, co-authors. NMDA: Noncompetitive N-methyl-D-aspartate.

Ketamine is a phencyclidine (PCP) derivative, which primarily acts as a noncompetitive N-methyl-D-aspartate (NMDA) receptor antagonist. It serves as a dissociative sedative that produces potent analgesia and amnesia by direct action on the cortex and limbic system while preserving spontaneous respiratory drive [13]. The mechanism in which ketamine provides analgesia or anesthesia is mainly glutamate-dependent. Glutamate is abundant in the central nervous system, and its release activates several pre- and postsynaptic receptors located on ion channels. NMDA receptors are glutamate receptors and are located in nearly all the cells in the central nervous system (CNS). When glutamate binds to the NMDA receptor, it causes membrane depolarization and ultimately activates the neuron (Figure 2). At anesthetic and dissociative dosing, ketamine works by blocking the NMDA receptor, thereby preventing the receptor from being activated by glutamate and decreasing the excitability of the neuron [14] (Figure 3).

**Figure 4:**
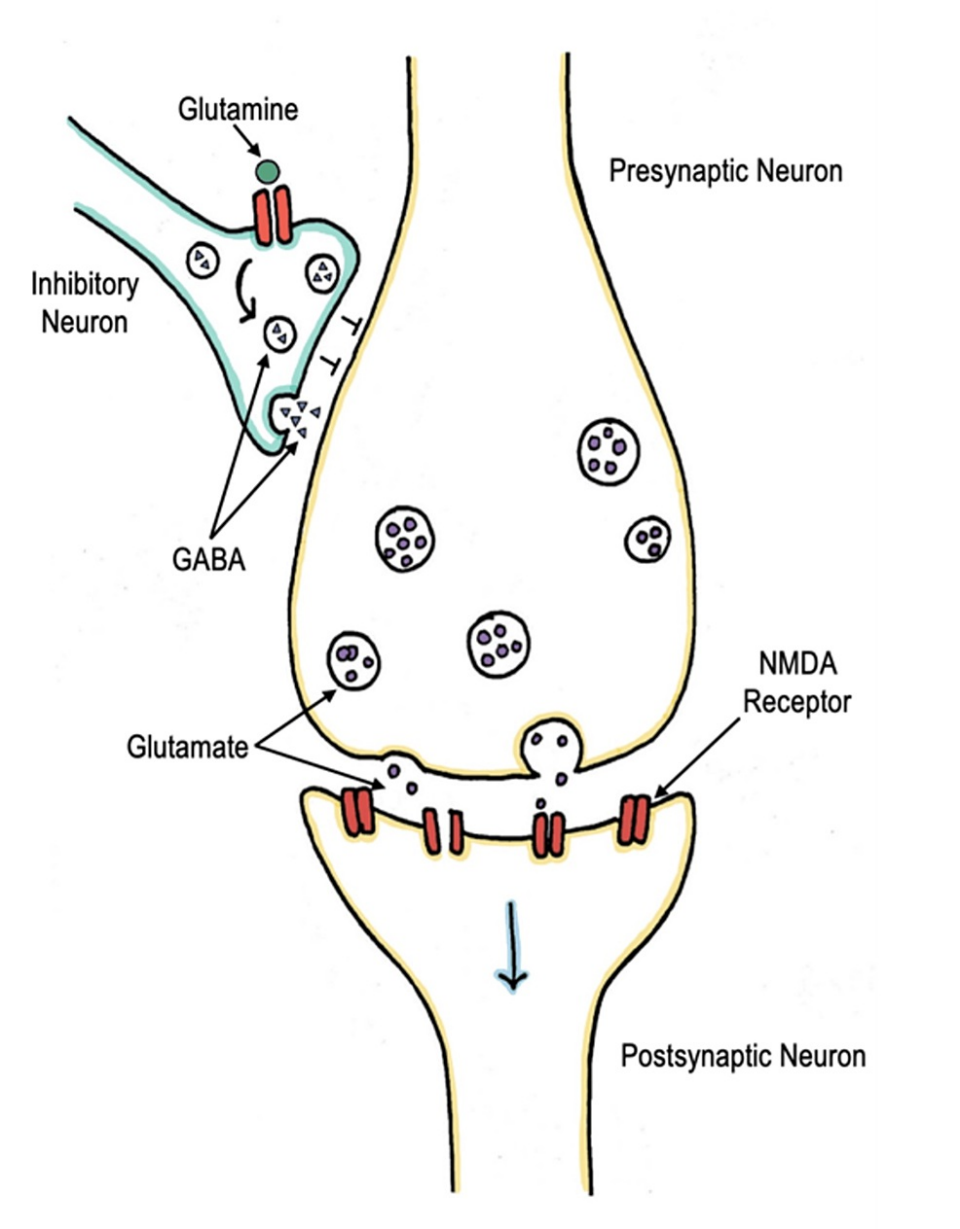
Mechanism of glutamine Glutamine can bind to the NMDA receptors on inhibitor neurons resulting in a release of GABA. GABA is an inhibiting neurotransmitter that when released inhibits the release of glutamate from the presynaptic neurons, resulting in no excitability of the postsynaptic neuron. Image credits: This drawing was created by Dana Nielsen and Kristen Reede, co-authors. NMDA: Noncompetitive N-methyl-D-aspartate; GABA: γ-amino-butyric acid.

**Figure 5:**
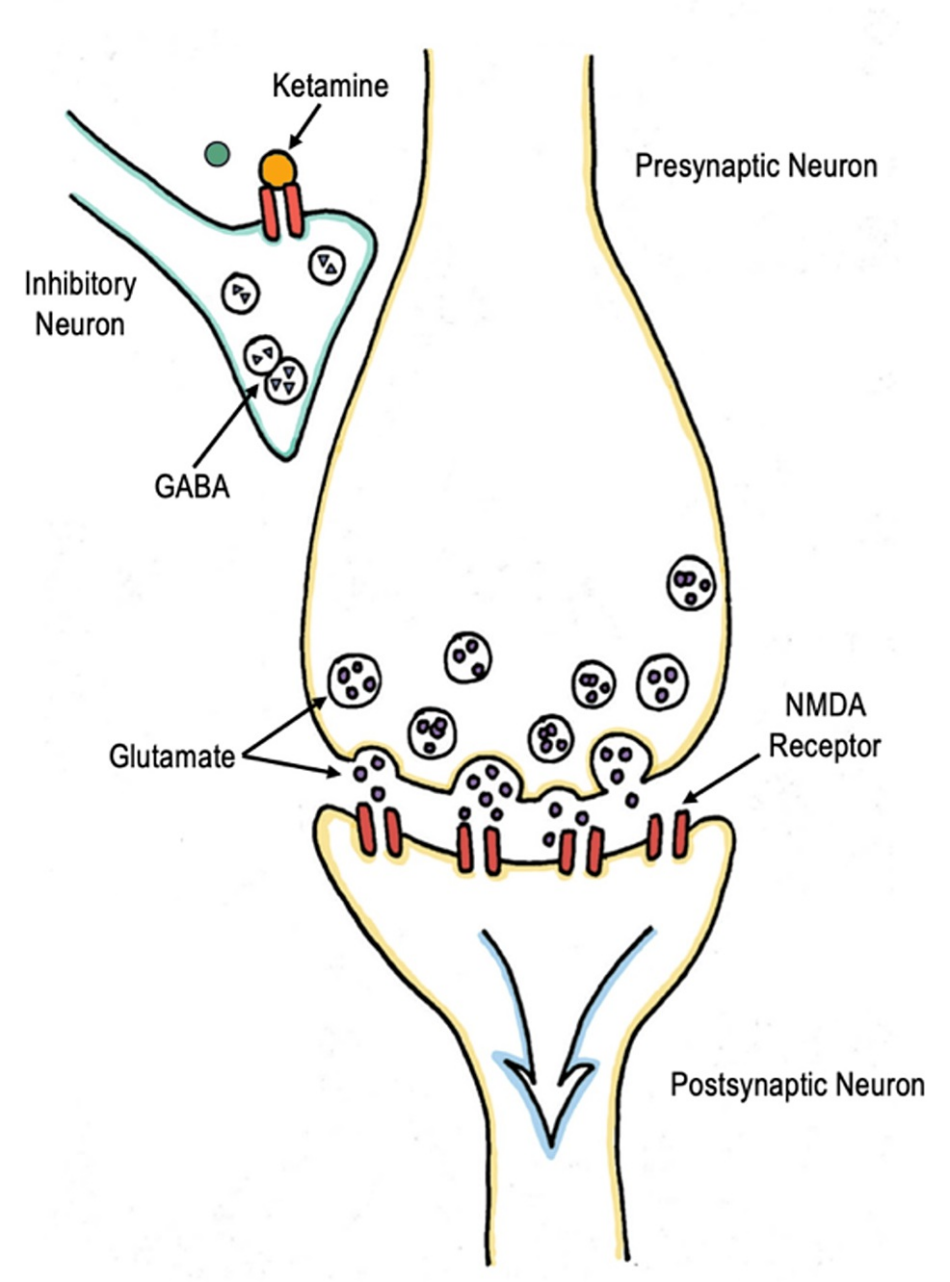
Mechanism of ketamine at subanesthetic dosing Ketamine blocks the NMDA receptors on inhibitory neurons, therefore decreasing the amount of GABA released. The presynaptic neuron is no longer inhibited, allowing the transmission of glutamate. This results in depolarization and excitability of the postsynaptic neuron. Image credits: This drawing was created by Dana Nielsen and Kristen Reede, co-authors. NMDA: Noncompetitive N-methyl-D-aspartate; GABA: γ-amino-butyric acid.

In subanesthetic doses, ketamine affects the release of γ-amino-butyric acid (GABA) on inhibitory interneurons. GABA is the most prevalent inhibiting neurotransmitter. In normal physiology, glutamine can bind to receptors on inhibitor neurons, causing a release of GABA from neurons. GABA inhibits the release of glutamine from the presynaptic neuron resulting in no excitability of the postsynaptic neuron (Figure 4). At subanesthetic doses, ketamine blocks the NMDA receptors on the inhibitory interneuron, therefore decreasing the amount of GABA that is released. The presynaptic neuron is no longer inhibited, allowing the transmission of glutamate, resulting in depolarization and firing of postsynaptic neurons (Figure 5). This mechanism allows the treatment of depression as it can facilitate synaptic plasticity and increase synaptogenesis. It also provokes a hyperadrenergic state due to an increased transmission of norepinephrine, dopamine, and serotonin into the circulation. As a result, some patients experience hypertension and tachycardia during the emergence of ketamine administration [14].

Pharmacokinetics

Ketamine is liposoluble resulting in a wide distribution throughout the body, including lipid-rich tissues and the central nervous system. This is advantageous for trauma patients as it can be administered in many ways including intravenous, intranasal, intramuscular, oral, and rectal. Ketamine is hepatically cleared through enzymes belonging to the cytochrome P450 system, which metabolizes ketamine into norketamine, which is further metabolized and excreted in bile and urine [10,14].

Because of the hepatic clearance, the effect of ketamine differs by the route being utilized. With intravenous and intramuscular administration, ketamine bioavailability reaches nearly 100% and 93%, respectively. This results in an expedited time to onset, less than one minute, with peak effect achieved at five minutes. Due to first-pass metabolism, ketamine administered orally has approximately 17%-29% bioavailability and reaches peak concentration in 20-30 minutes. Intranasal and intrarectal bioavailability are approximately 50% and 25%, respectively. Ketamine elimination half-life is two to three hours with a duration of action lasting between 10 and 25 minutes depending on the dose [14].

Ketamine produces rapid and reliable analgesia without causing prolonged sedation, which can be particularly useful in monitoring neurologic and hemodynamic changes in the trauma setting.

Ketamine in research

Ketamine is rapidly gaining acceptance in the management of pain in trauma patients as multiple studies have demonstrated its reliable efficacy and a wide margin of safety. Green et al.'s study was the first landmark study that changed the trajectory of ketamine use, which was published in 1990 in the *Annals of Emergency Medicine*. In this prospective, clinical trial, 108 children aged 14 months to 13 years underwent various procedures under ketamine sedation. It was noted that a single injection of ketamine achieved acceptable conditions in 97% of patients with 86% of patients not requiring adjunctive restraints or local anesthesia. Adequate sedation was produced within five minutes in 83% of patients, and the mean duration from injection to dischargeable recovery was 82 minutes (range: 30-175 minutes). One 18-month-old child vomited shortly after injection and experienced transient laryngospasm with cyanosis, but intubation was not required and there were no adverse sequelae. All other patients were able to maintain airway patency and independent respiration [15].

Since 1990, several studies have been published, which have demonstrated that ketamine inhibits the development of opiate tolerance, enhances opiate analgesia, and provides a safe and effective alternative adjunct to opioid analgesics [16-21]. The benefits of ketamine were further validated by a 2019 meta-analysis of seven articles by Yousefifard et al., which compared the pain management of trauma patients with prehospital administration of ketamine alone versus opioid analgesia (morphine or fentanyl) alone versus ketamine plus opioid analgesia [20]. Ketamine alone was less effective than administrating morphine or fentanyl in prehospital pain management (SMD = -0.56, 95% CI: -1.38 to 0.26, p = 0.117). However, co-administration of ketamine and morphine was considerably more effective than ketamine alone in alleviating pain (SMD = -0.62, 95% CI: -1.12 to -0.12, p = 0.010). Ketamine alone had fewer side effects than morphine alone (OR = 0.25, 95% CI: 0.11 to 0.56, p = 0.001), but when ketamine and morphine were given together, the risk of side effects increased by 3.68 times compared to when morphine was used alone (OR = 3.68, 95% CI: 199 to 6.82, p < 0.001) [22]. This concluded that ketamine alone is effective and provides fewer side effects compared to opioid analgesia but offers more effective pain control with combined opioid administration with the tradeoff of more side effects.

In addition to intravenous (IV) and intramuscular (IM) ketamine administration, intranasal ketamine also has proven to be safe and effective. It is gaining popularity due to its ease of use as it obviates the need for invasive routes of administration in patients without intravenous access, in patients who are seizing or combative, or in cases where obtaining intravenous lines is difficult and time-intensive. Furthermore, it provides a relatively painless alternative to IV or IM access, which is especially useful for children [23,24]. Intranasal (IN) route offers direct drug transport into the central nervous system circulation, thus largely bypassing hepatic first-pass metabolism and resulting in a rapid and predictable bioavailability compared to oral medications and some intramuscular medications [9,23,25].

Intranasal ketamine is shown to be as effective as intranasal narcotics in treating pain. Graudins et al. conducted a prospective, randomized controlled, double-blind equivalence study comparing IN ketamine to IN fentanyl for relief of moderate to severe pain due to limb injuries in children aged three to 13 years. The investigators compared IN ketamine (1 mg/kg) to IN fentanyl (1.5 μg/kg) in conjunction with ibuprofen (10 mg/kg) and assessed overall reduction in pain. There were similar pain reductions between groups at all points in addition to similar sedation scores between groups. There were more adverse events in the ketamine group (28/36; 78% vs. 15/37; 40%), although all were considered minor and insignificant [9,26]. Frey et al. compared the use of IN ketamine with IN fentanyl in pediatric patients aged eight to 17 years in the emergency department (ED) after an extremity injury with a double-blind, randomized, active control noninferiority study. They compared IN ketamine (1.5 mg/kg) with IN fentanyl (2 μg/kg) and assessed reduction in the visual analog scale. They determined that IN ketamine was non-inferior to fentanyl for pain reduction [27].

Intranasal ketamine has also been shown to reduce the number of narcotics used for acute traumatic pain. Bouida et al. performed a prospective, multicenter, randomized controlled, double-blind trial comparing IN ketamine to placebo saline in adult patients with moderate to severe acute limb trauma pain in the ED. The need for opioids was significantly lower in the IN ketamine group compared to the placebo group (17.2% vs 26.5%, P < 0.001). They also demonstrated that patients in the IN ketamine group required less non-opioid analgesics compared to the control counterpart (31.1% vs 39.6% respectively, P = 0.003) [28].

Ketamine in traumatic brain injuries

Traditionally, ketamine has been contraindicated in patients at risk for increased intracranial pressure (ICP). The theory was that ketamine administration increased cerebral blood flow and thus increased ICP. Shaprio et al. conducted a small study in 1972 to investigate how ketamine affects ICP in patients with abnormal cerebrospinal fluid dynamics and/or space-occupying lesions compared to neurologically normal patients [29]. The study involved administrating intravenous ketamine and measuring ICP before and after administration. This was completed on nine occasions in five patients. In the patients who had normal CSF pathways, the average ICP increase was 19.4 ± 6.9 mmHg. In the patients who had abnormal cerebrospinal fluid (CSF) pathways, the average ICP increase was 41.5 ± 16.6 mmHg. The results suggested that patients who have abnormal CSF dynamics or space-occupying lesions cannot compensate for the increase in cerebral blood flow as they have lost the buffering mechanism of displacing the CSF into other spaces. The increase in ICP can then result in a reduction of cerebral perfusion pressure, which is associated with cerebral hypoxia and tissue damage. It was therefore recommended not to use ketamine in patients with intracranial pathology. One of the downfalls of this study is that the investigators did not control for ventilation as changes in hypoxia and hypercarbia can also lead to reversible increases in ICP [29]. Since then, this finding has been repeatedly disproven.

In 1996, Kolenda and team performed a prospective study in which adult ventilated patients with moderate or severe traumatic brain injury (TBI) were prospectively allocated to receive treatment either with a combination of ketamine and midazolam or fentanyl and midazolam. The ICP was 2 mmHg higher in the ketamine group; however, this was not seen until day eight, and it was statistically insignificant. Interestingly, the ketamine group had higher cerebral perfusion pressure (CPP) by 8 mmHg. A similar prospective study was completed by Godoy et al. in which adult mechanically ventilated patients with severe TBI were allocated to receive treatment either with a combination of ketamine and midazolam or sufentanyl and midazolam. Again, there was no difference between the groups (mean: 19 vs 15.7 mmHg, p = 0.28) [30].

This phenomenon was also studied in children. Laws et al. studied the acute effects of ketamine on ICP and CPP in children with severe TBI. This was a prospective study of 82 ketamine administrations in 30 children with severe TBI. Ketamine was given for sustained ICP greater than 18 mmHg. They found that there was no increased ICP, and in fact, the ICP decreased after ketamine administration. One downfall of this study is that the Glasgow Coma Score (GCS) was not reported, thus limiting its generalizability [31].

In addition to several studies demonstrating that ketamine is safe to administer to TBI patients, there are also new findings that suggest ketamine has neuroprotective properties in adults. Multiple mechanisms have been described in which ketamine attenuates excitotoxicity, reduces calcium-mediated cell death process, reduces proinflammatory cytokine release by microglial cells, reduces microthromboses through inhibition of platelet aggregation, and upregulates the density of dendritic spines, leading to the sprouting of new neuronal synapses during the recovery period [32].

Considering the emerging evidence that ketamine does not increase ICP, the trauma foundation guidelines for the management of severe TBI in 2019 suggested reconsideration of ketamine use in that patient population. Clinicians should still use a pragmatic approach based on the clinical situation and their knowledge of the potential advantages and disadvantages before administering ketamine to TBI patients.

Ketamine dosing

There are several opportunities to utilize ketamine in traumatic and non-traumatic patients (Table 1). Ketamine can cause psychedelic effects that affect visual and auditory perceptions, mood, body awareness, and time. There may be symptoms such as floating, depersonalization, conscious dreams, or hallucinations. These effects are dose-dependent and more pronounced at higher concentrations of ketamine. Several dosing recommendations allow providers to produce either analgesic (Table 2) or dissociative results (Table 3).

**Table 1:** Indications for ketamine administration Credits: This table was created by Kristen Reede, a co-author.

Opportunities to utilize ketamine
Orthopedic closed reduction procedures
Realignment and splinting of fractures
Chest tube insertions
Skin closures on patients with low pain tolerance
Skin closures on children greater than three months old
Burn patients
Patients in shock
High-risk anesthesia patients
Patients in extreme distress
Facilitate extrication of patients
Facilitate transportation of patients

**Table 2:** Analgesic dosing recommendations Credits: This table was created by Kristen Reede, a co-author.

Route	Dosing
Intermittent intravenous	0.1-0.3 mg/kg; maximum dose of 30 mg at once, for a maximum of three doses
Intermittent intranasal	0.5-1 mg/kg; maximum dose of 40 mg
Continuous intravenous (adult)	0.1-0.4 mg/kg/h
Intramuscular (adult, non-weight based)	50 mg; repeat every 30-60 minutes until desired pain control is achieved or until nystagmus develops
Intermittent intraosseous (adult, non-weight based)	20 mg; slow push over 1 minute, repeat every 20 minutes until desired pain control is achieved

**Table 3:** Dissociative dosing recommendations Credits: This table was created by Kristen Reede, a co-author. IV: Intravenous; IM: Intramuscular.

Indication	Dosing
Procedural (IV)	1 mg/kg; maximum dose of 100 mg per dose
Induction of anesthesia (IV)	2 mg/kg; maximum dose of 200 mg per dose
Acute agitation (IV)	1-2 mg/kg
Acute agitation (IM)	3-5 mg/kg

Analgesia is generally achieved with lower dosing without inducing dissociative symptoms, while dissociation is achieved with higher dosing. Dissociation can be achieved by any dose greater than 0.5 mg/kg administered intravenously. Intramuscular doses greater than 5 mg/kg should be avoided as this could potentially cause agitation or psychosis without inducing sedation. Due to the volume limitation of the nasopharynx, intranasal doses greater than 40 mg per dose should also be avoided. If intranasal doses greater than 40 mg are administered, part of the dose will be delivered to the oropharynx and ingested orally, which may decrease effectiveness.

Administration

Before administration of ketamine, providers should ensure patients do not have any contraindications to the drug (Table 4). The administration of intravenous ketamine should always be completed in a slow and controlled fashion. Ketamine usually comes in 50 mg/ml or 100 mg/ml vials. Injections should never take less than one minute to administer. The full effect of the drug should be observed after five minutes. To facilitate a slow injection, ketamine can be diluted with a 5% dextrose solution. For example, if a dosing goal is 1 mg/kg for a 100 kg patient, two vials of 50 mg/ml will be required. Two vials, equaling 2 ml, can be diluted with 8 ml of 5% dextrose, and a total of 10 ml of ketamine/dextrose solution mixture should be administered over one minute. If 100 mg/ml vials are used, 1 ml of ketamine should be diluted with 9 ml of 5% dextrose, and the 10 ml solution would then be administered.

**Table 4:** Contraindications to ketamine use Credits: This table was created by Kristen Reede, a co-author.

When to avoid ketamine administration?
Systolic blood pressure (SBP) > 180 mmHg
Diastolic blood pressure (DBP) > 100 mmHg
Glasgow Coma Score (GCS) < 9
History of psychosis
Active manic or bipolar disorder
Active delirium, delusions, or hallucinations
Unstable heart disease
Current substance abuse of illicit substances
Active intoxication
Children less than three months
Pregnancy
Breastfeeding

During administration, vitals should be taken every 15 minutes for one hour, then every two hours for four hours, followed by every four hours if stable. Pain assessments should be done hourly. If dissociative dosing is desired, medical personnel should expect loss of consciousness in patients within 30-60 seconds after IV administration and two to four minutes after IM administration. Return of consciousness should occur 10-20 minutes after injection. Amnesia persists 60-90 minutes after recovery of consciousness. The systolic blood pressure (SBP) of patients may also increase by 20-40 mmHg.

Administration of the drug should stop immediately if any of the following events occur: SBP less than 85 mmHg, heart rate less than 60, respiratory rate less than 10 breaths per minute, suspected anaphylactic reaction, the patient experiences intolerable psychomimetic effects, or the patient requests to stop administration due to unpleasant side effects.

Side effects

Despite minimal effects on the respiratory drive, ketamine maintains the protective airway reflexes, which allows spontaneous ventilation to continue. However, when the dosage is very high or the medication is given too quickly, respiratory depression can occur. Ketamine is also responsible for bronchodilation, increased salivation, pulmonary vasodilation, and increased cardiac output, along with increased mean arterial pressure and heart rate. Other potential side effects of ketamine include dizziness, feeling of unreality, disorientation, nausea, vomiting, fatigue, headache, changes in hearing, mood change, hallucination, general discomfort, sedation, unpleasant taste, itchiness, rash, nystagmus, nasal irritation, euphoria, or agitation. Following infusion, emergence delirium occurs in 10%-20% of patients. Fifteen percent of patients experience disorientation, crying, or restlessness. Laryngospasms can occur in 0.4% of patients [10,27,28,33].

## Conclusions

Ketamine use in trauma management has a wide range of potential benefits, both physical and psychological. In a multifaceted approach to trauma care, ketamine plays a valuable role in amnesia, analgesia, and sedation, especially when other pain medications are contraindicated, ineffective, or unable to be administered due to insufficient IV access. It can provide rapid sedation to facilitate patient management and treatment in patients who are agitated, combative, or delirious. It is useful for short-duration procedural sedation such as for chest tube insertions, complex laceration repairs, or reductions in fractures or joint dislocations. It is particularly helpful when the use of opioid medication is risky due to respiratory depression or hemodynamic instability. Lastly, ketamine can be used as an induction agent in patients who require emergency airway management and intubation. As stated in this article, the range of utilization of ketamine is substantial as it provides reliable and effective amnesia, sedation, and analgesia while preserving respiratory drive and maintaining cardiovascular stability and the patients’ protective reflexes. To further expand the use of ketamine in diverse clinical settings, continued research and careful evaluation are essential.
